# Supplementary material for: Gut microbiome features associate with immune checkpoint inhibitor response in individuals with non-melanoma skin cancers: an exploratory study
Source: Microbiol Spectr. 2025 Feb 3;13(3):e02559-24. doi: 10.1128/spectrum.02559-24 (PMC11878019; doi:10.1128/spectrum.02559-24)
Supplement: Table S1 — Participant characteristics. [file spectrum.02559-24-s0001.docx]

| **Characteristic** | **BCC** N = 5*^1^* | **MCC** N = 5*^1^* | **SCC** N = 11*^1^* |
| --- | --- | --- | --- |
| Age [yrs] (sd) | 77 (13) | 69 (1) | 74 (10) |
| Sex (%) |  |  |  |
| Female | 3 (60%) | 1 (20%) | 4 (36%) |
| Male | 2 (40%) | 4 (80%) | 7 (64%) |
| Treatment (%) |  |  |  |
| Avelumab(anti-PD1/PDL1) | 0 (0%) | 1 (20%) | 0 (0%) |
| Cemiplimab(anti-PD1/PDL1) | 0 (0%) | 0 (0%) | 7 (64%) |
| Nivolumab(anti-PD1/PDL1) | 4 (80%) | 1 (20%) | 0 (0%) |
| Nivolumab(anti-PD1/PDL1)+Ipilimumab(anti-CTLA4) | 1 (20%) | 0 (0%) | 0 (0%) |
| Pembrolizumab(anti-PD1/PDL1) | 0 (0%) | 3 (60%) | 4 (36%) |
| ICI Response (%) |  |  |  |
| Nonresponder/NR | 1 (20%) | 1 (20%) | 4 (36%) |
| Other (radiotherapy, organ transplant, unevaluable) | 0 (0%) | 1 (20%) | 3 (27%) |
| Responder/R | 2 (40%) | 3 (60%) | 4 (36%) |
| Stable_Disease/SD | 2 (40%) | 0 (0%) | 0 (0%) |
| Prior Treatment |  |  |  |
| Immune checkpoint inhibitor | 0 (0%) | 1 (20%) | 0 (0%) |
| Targeted | 3 (60%)^2^ | 0 (0%) | 3 (27%)^3^ |
| Immune checkpoint inhibitor +Targted | 1 (20%)^2^ | 0 (0%) | 0 (0%) |
| No prior treatment | 1 (20%) | 4 (80%) | 8 (73%) |
| Total Fecal Samples | 19 | 22 | 27 |
| *^1^*Mean (SD); n (%); Sum; ^2^cetuximab; ^3^vismodegib | | | |
