# Supplementary material for: Gut microbiome features associate with immune checkpoint inhibitor response in individuals with non-melanoma skin cancers: an exploratory study
Source: Microbiol Spectr. 2025 Feb 3;13(3):e02559-24. doi: 10.1128/spectrum.02559-24 (PMC11878019; doi:10.1128/spectrum.02559-24)
Supplement: Supplemental material — Fig. S1 to S3; Methods. [file spectrum.02559-24-s0002.docx]

**Supplemental Material**

Table of Contents

[Methods 1](#_Toc163821647)

[Study design, eligibility criteria and participants 1](#_Toc163821648)

[Sample and data collection. 2](#_Toc163821649)

[Fecal DNA extraction and sequencing. 2](#_Toc163821650)

[Analysis of 16S rRNA amplicon taxonomic data. 2](#_Toc163821651)

[Fecal metabolite extraction, data acquisition, and data processing. 2](#_Toc163821652)

[Statistical analysis. 3](#_Toc163821653)

[Supplemental Figures 4](#_Toc163821654)

[Figure S1 Alpha Diversity across all tumor types (MCC, BCC and CSCC combined) 4](#_Toc163821655)

[Figure S2 Principal Coordinate Analysis of first sample across all tumor types (MCC, BCC and CSCC combined) 4](#_Toc163821656)

[Figure S3 Alpha Diversity in SCC 5](#_Toc163821657)

[Reference 5](#_Toc163821658)

# Methods

Study design, eligibility criteria and participants**.**

The study protocol and all amendments were approved by the Institutional Review Board of Johns Hopkins University (Johns Hopkins Medicine Institutional Review Board). Written informed consent was obtained from all participants; all individuals were enrolled from 10/20/2015 to 7/14/2020 with data lock on 10/11/2023. Eligibility criteria included the following: age >18 and treatment with an immune checkpoint inhibitor at Johns Hopkins Hospital, Sibley Memorial Hospital, or Johns Hopkins Howard County Medical Center.

68 fecal samples were collected from 21 individuals with NMSC either before the treatment start date, during, and/or after treatment. Based on Best Overall Response according to RECIST.v1.1^1^, individuals with progressive disease were defined as non-responders (NR) and individuals with a partial or complete response were defined as responders (R). Additional categories included individuals with stable disease (SD), and individuals treated with radiotherapy or organ transplant were classified as other/unevaluable.

Sample and data collection.

All fecal samples were collected by participants at home as either fresh stool or placed into an OMNIgene GUT (DNA Genotek, OM-200) tube. Fresh stool was stored at 4⁰C, brought to clinic within 48 hours, and aliquoted in a laminar flow hood. OMNIgene GUT tubes were mailed or brought to the lab within 60 days, vortexed, and aliquoted in an anaerobic hood. All sample aliquots were stored at -80⁰C. All samples are indicated in **Table S2** along with associated clinical data, curated by the treating physician and study coordinators

Fecal DNA extraction and sequencing.

Approximately 80mg of fresh stool or 250ul of OMNIgene GUT stool sample were transferred to BashingBead Lysis Tubes (Zymo, 0.1/0.5 mm beads) with bead bashing buffer. All samples underwent mechanical lysis using a Mini-Beadbeater-96 (Biospec Products) at 2400 rpm for 60 seconds, 3 cycles. The remainder of the DNA extraction was performed using the Quick-DNA Fecal/Soil Microbe Kit (Zymo, 96 well plate). DNA quantity and purity were measured using a Nanodrop (Thermo Scientific). All sequencing was performed by the CHOP Microbiome Center as previously described^2^.

Analysis of 16S rRNA amplicon taxonomic data.

Raw sequencing files were demultiplexed and assigned to operational taxonomic units (OTUs) using Resphera Insight (v.2.2) as previously described^2^ with an abundance threshold of 0.01%. Alpha diversity measures were calculated using R package phyloseq (v1.42.0). Beta diversity was calculated based on Bray-Curtis distance using R package Vegan (v2.6.4). Functional profiles were predicted using PICRUSt2 (v.1.0.0)^3^ based on Metacyc databases^4^. OTUs to the same distinct species were aggregated prior to final analysis.

Fecal metabolite extraction, data acquisition, and data processing.

For each sample, 80mg of fecal material was transferred to a sterile individual screw cap tube with a 3mm metal bead (Qiagen) in a laminar flow hood on ice. Metabolites were extracted in ice-cold 80% methanol and homogenized by vortex. Samples centrifuged for 1 minute at 10,000xg at 4⁰C and extracted supernatant was stored at -80⁰C. Samples were analyzed at General Metabolics (Cambridge, MA). For these analyses, the extracts were diluted 1:225 with 80% (v/v) methanol in water solution. Metabolome profiles of the sample extracts were acquired using flow-injection mass spectrometry^5^. Samples were run on an Agilent 6550 iFunnel LC-MS Q-TOF mass spectrometer in tandem with an MPS3 autosampler (Gerstel) and an Agilent 1260 Infinity II quaternary pump. The running buffer was 60% isopropanol in water (v/v) buffered with 1 mM ammonium fluoride. Hexakis (1H, 1H, 3H-tetrafluoropropoxy)-phosphazene) (Agilent) and 3-amino-1-propanesulfonic acid (HOT) (Sigma Aldrich) were added to the running buffer to serve as lock masses. The isocratic flow rate was set to 0.150 mL/min. The instrument was run in 4GHz High Resolution, negative ionization mode. Mass spectra between 50 and 1,000 m/z were collected in profile mode. 5 uL of each sample were injected twice, consecutively, within 0.96 minutes to serve as technical replicates. A pooled study sample (pSS) was prepared by pooling 5 uL of each sample together and was injected periodically throughout the batch for additional quality control. Samples were acquired randomly within plates. Raw profile data were centroided, merged, and recalibrated using MATLAB software. Putative annotations were generated based on compounds contained in Human Metabolome Database^6^, Kyoto Encyclopedia of Genes and Genomes (KEGG)^7^, and Chemical Entities of Biological Interest (ChEBI)^8^ using both accurate mass per charge (tolerance 0.001 m/z) and isotopic correlation patterns. Metabolites that were below the bottom 10% peak intensity are removed for further analysis.

## Statistical analysis.

Statistical analyses were performed using R v4.2.2. Linear discriminant analysis Effect Size (LEfSe) v1.1.0110 was used to identify significant bacterial taxa and predicted functional Metacyc pathways associated with participant clinical responses to ICI therapy. Student's t test was used to identify significant untargeted metabolomics data associated with participant responses using MetaboAnalyst 5.0 web software. All significant features are selected using a cutoff p < 0.1. Significant metabolites associated with R and NR were submitted to MetaboAnalyst 5.0 web software to perform pathway enrichment analysis using KEGG. To visualize the variance of bacterial taxa, principal co-ordinates (PCoA) analysis was performed using R package ade4 (v1.7.22). Permutational Multivariate Analysis of Variance (PERMANOVA) analysis was performed using R package Vegan (v2.6.4). All the visualization is plotted using R package ggplot2 (v3.4.4), ggpubr (v0.6.0), swimplot (v1.2.0), and pheatmap (v1.0.12).

# Supplemental Figures

## Figure S1 Alpha Diversity across all tumor types (MCC, BCC and CSCC combined)

***
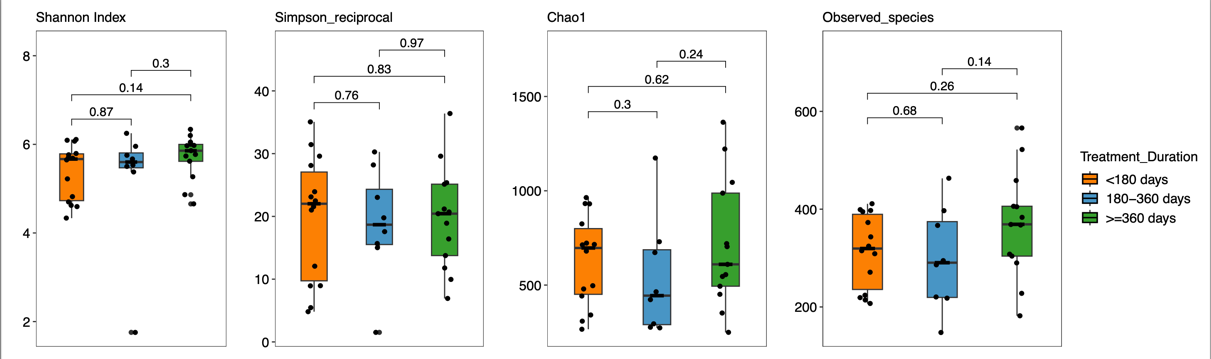
***

***Fig S1:*** Longitudinal alpha diversity, measured by Shannon diversity index, Simpson reciprocal index, Chao1 index, and observed species is plotted for fecal samples (n = 35 samples from 9 individuals) collected at different treatment timepoints within R across all tumor types. Statistics by Mann-Whitney test. The colored boxes represent the median (line inside the box) and the 25% to 75% interquartile range (bottom and top edge). The upper and lower whiskers represent 95% confidence intervals.

## Figure S2 Principal Coordinate Analysis of first sample across all tumor types (MCC, BCC and CSCC combined)


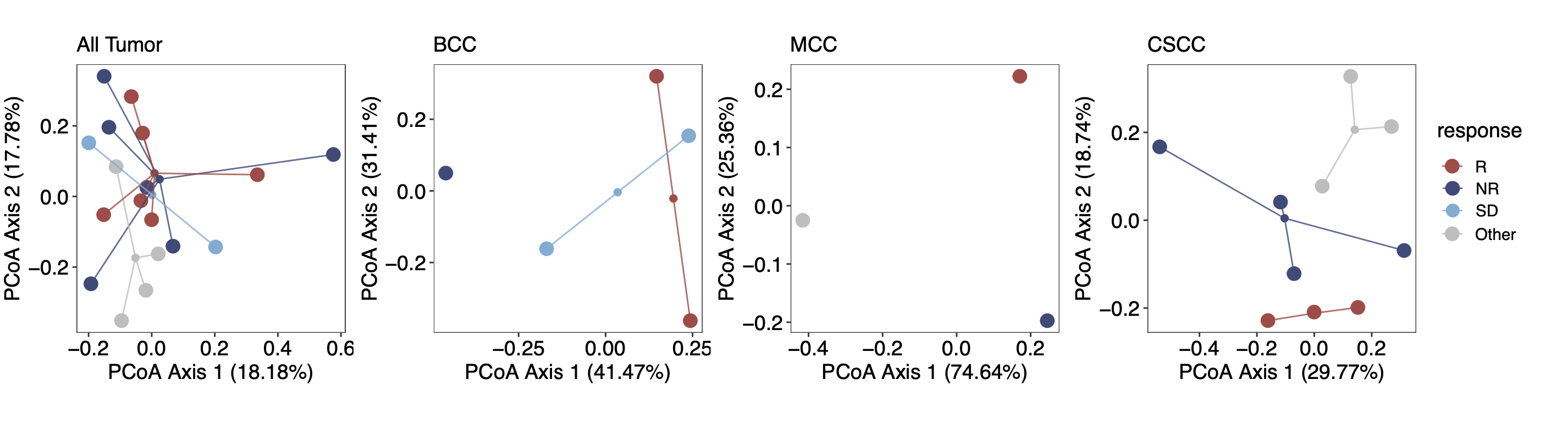
***Fig S2:*** Principal Coordinate Analysis (PCoA) of the first fecal sample of each individual (n=18, each with one fecal sample) using the Bray-Curtis distance metric across all tumor types (left), as well as within BCC (n=5, each with one fecal sample), MCC (n=3, each with one fecal sample), and CSCC (n=10, each with one fecal sample). Color displays ICI responses: non-responder (NR), blue; responder (R), red; other, grey.

## Figure S3 Alpha Diversity in CSCC


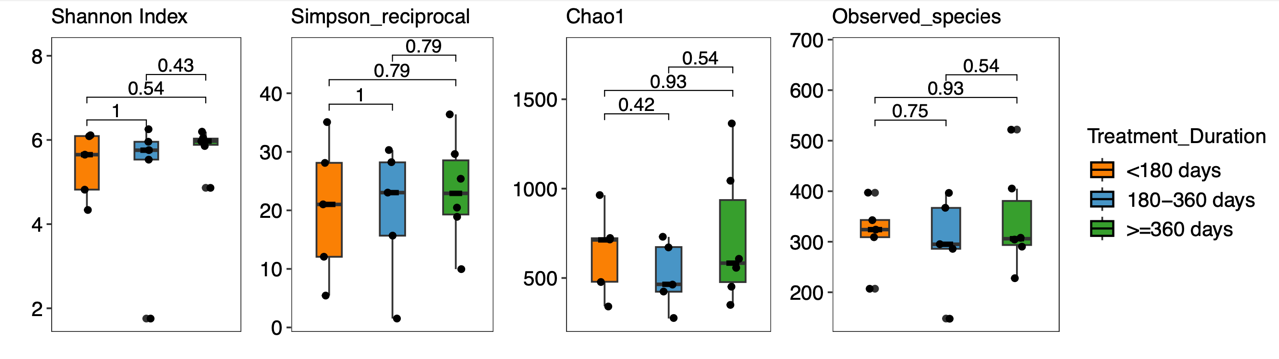


***Fig S3:*** Longitudinal alpha diversity, measured by Shannon diversity index, Simpson reciprocal index, Chao1 index, and observed species is plotted for fecal samples (n = 16 samples from 4 individuals) collected at different treatment timepoints within responder (R) in CSCC. Statistics by Mann-Whitney test. The colored boxes represent the median (line inside the box) and the 25% to 75% interquartile range (bottom and top edge). The upper and lower whiskers represent 95% confidence intervals.

# Reference

1. Eisenhauer, E. A. *et al.* New response evaluation criteria in solid tumours: Revised RECIST guideline (version 1.1). *European Journal of Cancer* **45**, 228–247 (2009).

2. Shaikh, F. Y. *et al.* Murine fecal microbiota transfer models selectively colonize human microbes and reveal transcriptional programs associated with response to neoadjuvant checkpoint inhibitors. *Cancer Immunol Immunother* **71**, 2405–2420 (2022).

3. Douglas, G. M. *et al.* PICRUSt2 for prediction of metagenome functions. *Nat Biotechnol* **38**, 685–688 (2020).

4. The MetaCyc Database - PMC. https://www.ncbi.nlm.nih.gov/pmc/articles/PMC99148/.

5. Fuhrer, T., Heer, D., Begemann, B. & Zamboni, N. High-throughput, accurate mass metabolome profiling of cellular extracts by flow injection-time-of-flight mass spectrometry. *Anal Chem* **83**, 7074–7080 (2011).

6. Wishart, D. S. *et al.* HMDB 5.0: the Human Metabolome Database for 2022. *Nucleic Acids Res* **50**, D622–D631 (2022).

7. Kanehisa, M. & Goto, S. KEGG: kyoto encyclopedia of genes and genomes. *Nucleic Acids Res* **28**, 27–30 (2000).

8. Hastings, J. *et al.* The ChEBI reference database and ontology for biologically relevant chemistry: enhancements for 2013. *Nucleic Acids Res* **41**, D456–D463 (2013).
