# Supplementary material for: Gut microbiome features associate with immune checkpoint inhibitor response in individuals with non-melanoma skin cancers: an exploratory study
Source: Microbiol Spectr. 2025 Feb 3;13(3):e02559-24. doi: 10.1128/spectrum.02559-24 (PMC11878019; doi:10.1128/spectrum.02559-24)
Supplement: Supplemental table legends — Legends for Tables S1 to S4. [file spectrum.02559-24-s0003.docx]

**Table S1 Participants Characteristics**

**Table S1:** Clinical characteristics for 21 individuals with non-melanoma skin cancer (n = 5 BCC, basal cell carcinoma; 5 MCC, merkel cell carcinoma; 11 CSCC, cutaneous squamous cell carcinoma).

**Table S2 Alpha Diversity, Clinical Characteristics and Relative Abundance Table**

**Table S2:** Alpha diversity, Clinical Characteristics and relative abundance of microbial taxa derived from 16S rRNA sequencing of 68 fecal samples (n= 19 collected for 5 BCC individuals, basal cell carcinoma; n= 22 collected for 5 MCC individuals, merkel cell carcinoma; n= 27 collected for 11 CSCC individuals, cutaneous squamous cell carcinoma).

**Table S3 Untargeted Metabolomics Data**

**Table S3:** Untargeted metabolomics data collected and annotated using the first fecal sample of each individual with CSCC collected within 180 days (n=6 individuals, 3 responders (R) and 3 non-responders (NR), each with one fecal sample).

**Table S4 Differential Metabolomics in CSCC**

**Table S4:** Differential metabolites associated with immunotherapy responses identified by t-test (p< 0.1) using the first fecal sample of each individual with CSCC collected within 180 days (n=6 individuals, 3 responders (R) and 3 non-responders (NR), each with one fecal sample).
